# Leucine Supplementation Counteracts the Atrophic Effects of HDAC4 in Rat Skeletal Muscle Submitted to Hindlimb Immobilization

**DOI:** 10.1002/mus.28411

**Published:** 2025-04-04

**Authors:** Paula K. N. Alves, André Cruz, William J. Silva, Afonso M. Melazzo, Siegfried Labeit, Volker Adams, Anselmo S. Moriscot

**Affiliations:** ^1^ Department of Anatomy Institute of Biomedical Sciences, University of Sao Paulo Sao Paulo Brazil; ^2^ Faculty for Clinical Medicine Mannheim of the University of Heidelberg, Institute for Integrative Pathophysiology Mannheim Germany; ^3^ Myomedix GmbH Neckargemund Germany; ^4^ Laboratory of Molecular and Experimental Cardiology TU Dresden, Heart Center Dresden Dresden Germany

**Keywords:** HDAC4, hindlimb immobilization, leucine, rats, skeletal muscle

## Abstract

**Introduction/Aims:**

We previously demonstrated that leucine supplementation significantly reduces histone deacetylase 4 (HDAC4) expression induced by hindlimb immobilization, thereby attenuating the increase in HDAC4 protein levels and nuclear accumulation. In this study, we investigated the impact of supraphysiological HDAC4 levels on skeletal muscle and the inhibitory potential of leucine in this scenario.

**Methods:**

A total of 64 male Wistar rats were used in this study and subjected to electroporation of the soleus muscle with or without a plasmid overexpressing HDAC4 mRNA, followed by hindlimb immobilization and leucine supplementation (1.35 g/kg) for 7 days.

**Results:**

Our findings revealed that HDAC4 overexpression alone led to soleus atrophy, resulting in a 23% decrease in mass, a 31% reduction in whole muscle cross‐sectional area (CSA), and a 17% decrease in fiber CSA. These reductions were further exacerbated by hindlimb immobilization, with decreases of 50%, 46%, and 34%, respectively. Moreover, leucine supplementation protected against soleus atrophy and preserved soleus fiber CSA by 17%. This protective effect was accompanied by a 57% reduction in HDAC4‐positive nuclear localization in immobilized rats overexpressing HDAC4.

**Discussion:**

Our results indicate that HDAC4 forced expression can alone induce skeletal muscle atrophy. In addition, our results indicate that leucine is dominant in blocking HDAC4 signaling and highlight the use of this amino acid as a therapeutic tool in conditions involving skeletal muscle atrophy.

## 1 Introduction

Physical inactivity, such as in chronic illness or limb immobilization, disrupts muscle homeostasis balance, leading to increased protein breakdown and atrophy [1, 2], which is a complex process involving post‐transcriptional modifications, autophagy, proteolysis activation, and gene transcription regulation, including the activation of histone deacetylases. Histone deacetylase 4 (HDAC4) is predominantly found in the nuclei of muscles undergoing denervation [3], resulting in increased transcription of key ubiquitin‐proteasome mediators of proteolysis (Atrogin‐1 and MuRF1), which contribute to muscle atrophy [4, 5].

HDAC4 is a key protein involved in skeletal muscle atrophy and has been extensively studied in denervation models and immobilization‐induced atrophy [4, 6, 7, 8]. Atrophic conditions significantly increase HDAC4 gene and protein levels, leading to its nuclear translocation and the upregulation of myogenin [3, 9]. Studies have confirmed the HDAC4/myogenin axis through knockdown and overexpression experiments, suggesting a positive feedback loop [3, 5, 9]. Myogenin also activates the promoters of Atrogin‐1 and MuRF1, linking the HDAC4/myogenin pathway to ubiquitin‐dependent proteolysis in skeletal muscle [4].

Various studies have used HDAC4 inhibition models, such as muscle‐specific knockout (KO), pharmacological inhibitors, and shRNAi, to explore its impact on muscle performance. It has been shown that HDAC4 KO animals had 20% less muscle loss in the denervation model [4]. Similar results were obtained by either HDAC pharmacological inhibition [10] or by the use of shRNAi [11].

The effects of HDAC4 overexpression on skeletal muscle remain largely unexplored, with only a few studies addressing the overexpression effects of its downstream component, myogenin [4, 12, 13]. Investigating HDAC4 ectopic expression could provide valuable insights into its role in skeletal muscle atrophy at supraphysiological levels, improving our understanding of its involvement in muscle physiology and pathology and leading to more effective therapeutic strategies targeting HDAC4.

Our prior research showed that leucine supplementation reduces HDAC4 and myogenin expression, preventing muscle atrophy during immobilization and improving muscle performance [6]. Leucine also decreased HDAC4 nuclear localization, both in skeletal muscle [6] and the myocardium of HFpEF rats [14], suggesting that HDAC4 inhibition plays a role in leucine's anti‐atrophic effects. Nonetheless, the effects of HDAC4 in skeletal muscle and whether leucine could still counteract atrophy are unknown.

In this study, we examined the effects of supraphysiological HDAC4 levels and leucine supplementation in immobilized rats.

## 2 Methods

Descriptions of immunofluorescence and Western blotting methodologies, along with uncropped western blot membranes for both main and Supporting Information figures, are provided in the Supporting Information.

### 2.1 Experimental Animals

All animals were maintained in a bioterium with controlled temperature (24°C ± 1°C, 12‐h light–dark cycle) and standard food (Nuvilab CR‐1, Nuvital‐Quimtia, Brazil) and water were offered ad libitum.

In the first set of experiments, 24 animals were divided into four groups: Control group (7 days, *n* = 6); Immobilized group (Imm 7d, *n* = 6); Leucine group (Leu 7d, *n* = 6); and immobilized + leucine group (Imm + Leu 7d, *n* = 6) (details presented in Figure S1A).

In the second set of experiments, 16 animals were divided into four groups: Control groups for 4 and 8 days after electrotransfer (EV 4D and 8D, *n* = 4 per group) and pCMVHDAC4 for 4 and 8 days after electrotransfer (pCMVHDAC4 4D and 8D, *n* = 4 per group) (details presented in Figure S1B).

In the third set of experiments, 20 animals were divided into four groups: EV group (*n* = 5); pCMVHDAC4 (*n* = 5); Imm/pCMVHDAC4 (*n* = 5); Imm + Leu/pCMVHDAC4 (*n* = 5) (details presented in Figure S1C).

Leucine (L‐Leucine, Sigma—#L8000) was orally administered once a day at a (1.35 g/kg body mass, adapted from Baptista et al. and Pereira et al. [15, 16]), starting 3 days prior to immobilization (preloading).

The animals were euthanized by cervical dislocation, and the soleus muscle was harvested, weighed, and transversely cut into two parts: One half was immersed in hyper‐cooled isopentane and then snap‐frozen in liquid nitrogen for histological analysis, and the other half was snap‐frozen in liquid nitrogen for gene and protein expression analysis. For storage, the samples were kept at −80°C.

This study was approved by the Institute of Biomedical Sciences ethics committee (#2034200718) and followed the Code of Practice for the Housing and Care of Animals Used in Scientific Procedures.

### 2.2 HindLimb Immobilization

Rats submitted to immobilization were anesthetized using a ketamine and xylazine cocktail (100 and 10 mg/kg, respectively), and then prepared for immobilization procedures. The left hindlimb was fixed in a full plantar flexion position with plaster and then covered in a steel net [17].

### 2.3 Muscle Transfection by Electrotransfer

For the electroporation procedure, animals were anesthetized by a xylazine/ketamine cocktail (100 and 10 mg/kg, respectively). The soleus muscle was isolated, and then hyaluronidase (40 U, Sigma #H3506, Neustadt, Germany) was applied directly to the muscle. After 30 min, muscles were injected with 50 μL of the HDAC4 expression vector (1.0 μg/μL, pCMVHDAC4, Origene CAT#: RR217458, Rockville, MD, USA) or the empty vector (EV) and an electric pulse generator delivered a burst of pulses (6 pulses of 20 ms with 980 ms pause, 25 V).

### 2.4 Muscle Function Analysis

The muscle function analysis was performed as described previously [18]. In summary, the left soleus was dissected and mounted in a Krebs–Henseleit buffer‐filled organ bath (AVS system, Aqda/Ancad software, Sao Carlos, Brazil). Muscle function was assessed by platinum electrodes stimulating the muscle with a supramaximal current (700 mA, 500 ms train duration, 1.0 ms pulse width, AVS systems, Sao Carlos, Brazil). The muscle bundle was set at an optimal length (Lo) and after a 15‐min adaptation period, a force‐frequency protocol was performed at 1, 15, 30, 50, 80, 120, and 150 Hz, separated by 1‐min rest intervals. Maximum tetanic force was obtained by force‐frequency protocol.

### 2.5 Cross‐Sectional Area (CSA) Analysis

Muscles were transversely sectioned (10 μm thick) using a cryostat (Leica CM1850 UV, Wetzlar, Germany) and then stained with laminin by the immunofluorescence method. Photomicrographs were obtained by Axio Scope.A1 (Carl Zeiss Microscopy GmbH, Göttingen, Germany). The CSA of muscle fibers was obtained using the software ImageJ (v. 1.45 s, National Institutes of Health). The quantifications were conducted in a blinded manner, ensuring that the person who quantified was unaware of the experimental groups.

### 2.6 RNA Extraction and Real‐Time PCR

Muscle samples (± 20 mg) were homogenized and Trizol was used to isolate total RNA (Invitrogen) following the manufacturer's recommendations. The pellets were resuspended in ultrapure water, and RNA concentrations were determined at 260 nm absorbance (Eppendorf). The 260/280 ratio was used as a reference for RNA purity, and RNA integrity was verified on agarose denaturing gels stained with ethidium bromide.

cDNA synthesis was performed with 2 μg of total RNA and was used to perform real‐time PCR (with EvaGreen qPCR Supermix, Solis Biodyne, 200 nM of each primer, Table S1). PCR was run at 95°C for 12 min, followed by 40 cycles of 95°C for 15 s, 60°C for 30 s, and 72°C for 30 s. Cyclophilin A expression was used as a housekeeping gene.

### 2.7 Statistical Analysis

Multiple comparisons were established using either one‐way ANOVA followed by Tukey's posthoc test (for parametric data) or Kruskal‐Wallis test followed by Dunn's posthoc test (for non‐parametric data). Data are presented as mean ± SEM. GraphPad Prism 7.0 (Boston, MA/USA) was used for analysis, and *p* < 0.05 was considered significant.

## 3 Results

### 3.1 Leucine Supplementation Protects Soleus Muscle From Immobilization‐Induced Atrophy

**FIGURE 1:**
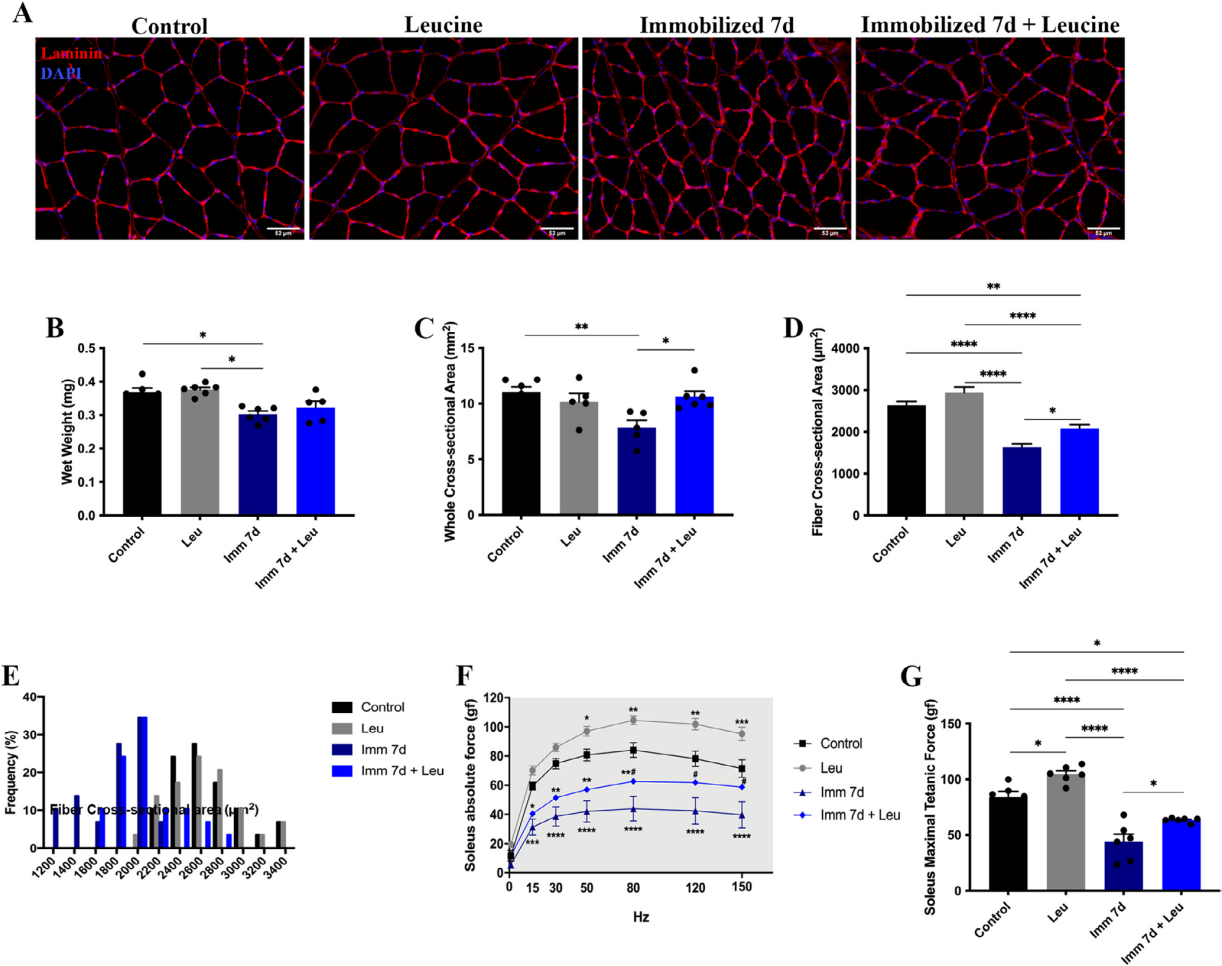
Analysis of leucine supplementation on soleus muscle morphometry and function after 7 days of hind limb immobilization. (A) Representative immunofluorescence photomicrographs of soleus muscle after 7 days of hind limb immobilization, laminin (red) and DAPI (blue, used for identification of nuclei) (scale bar 52 μm). (B) Average of soleus muscle wet weight, (C) whole muscle cross‐sectional area (CSA) and (D) fiber CSA. (E) Fiber size distribution from control, supplemented with leucine, immobilized, and immobilized supplemented with leucine after 7 days. (F) Analysis of soleus absolute force (*p < 0.05; **p < 0.01; ***p < 0.001; ****p < 0.0001 vs. Control; #*p* < 0.05 vs. Imm 7d) and (G) soleus maximum tetanic force. Data are expressed as mean ± SEM. Statistical analysis included the two‐way ANOVA test followed by Tukey's *post hoc*. *p < 0.05; **p < 0.01; ****p < 0.0001 (*n* = 5–6 per group).

After 7 days of hindlimb immobilization, the Imm 7d group showed a 16% decrease in soleus wet weight compared to the control group and a 19% decrease compared to the leucine group (Figure 1B). We also observed a 30% decrease in the whole muscle cross‐sectional area in the Imm 7d group compared to the control group, an effect that was reversed by leucine supplementation (27% protection) compared to Imm 7d (Figure 1C). We also measured fiber CSA and found a 39% decrease in the Imm 7d group compared to the control group, with leucine supplementation attenuating this by 22% compared to Imm 7d (Figure 1D). The frequency distribution graph of fiber cross‐sectional area (Figure 1E) showed that smaller fibers had a higher frequency in the Imm 7d group. As expected, leucine supplementation was able to shift this distribution toward larger fibers (Figure 1E).

In terms of soleus muscle function, absolute force decreased by approximately 50% across the force/frequency curve in the Imm + 7d group, with leucine supplementation reducing this decrease by 24% starting at 80 Hz compared to Imm 7d (Figure 1F). Leucine alone induced a 25% increase in soleus muscle absolute force compared to the control group (Figure 1F). The maximum tetanic force (measured at 80 Hz) showed a clear 48% reduction in the Imm 7d group compared to the control group, with leucine supplementation providing a protective effect of 25% compared to Imm 7d (Figure 1G). There were no significant differences in the soleus specific force analysis.

### 3.2 Overexpression of HDAC4 Induces Soleus Muscle Atrophy, Which Is Attenuated by Leucine Supplementation

We performed electrotransfer of a plasmid containing HDAC4 mRNA into rat soleus muscle. Information about the plasmid map, hybridization sites of primers, and transfection efficiency is presented in Figure S2.

In a preliminary experiment, we determined the optimal time to implement leucine supplementation and hindlimb immobilization in this set of experiments (details in Figure S3). We selected two time points: 4 days (pCMVHDAC4‐4D group) and 8 days (pCMVHDAC4‐8D group) after electrotransfer (Figure S3). Our results demonstrated that HDAC4 overexpression is sufficient to drive soleus muscle atrophy. Morphometric analysis of the muscle revealed a reduction in soleus mass (10%), whole muscle CSA (24%), and fiber CSA (43%) only after 8 days of electrotransfer when compared to the EV group (Figure S3B,D,F, respectively). Furthermore, analysis of HDAC4 protein expression revealed a 2‐fold and 3.5‐fold increase at 4 and 8 days post‐electroporation, respectively (Figure S3K). In consequence of the consistent atrophic results and higher HDAC4 protein expression, we selected the 8‐day post‐electroporation time point to carry out the experiment of hindlimb immobilization and leucine supplementation.

**FIGURE 2:**
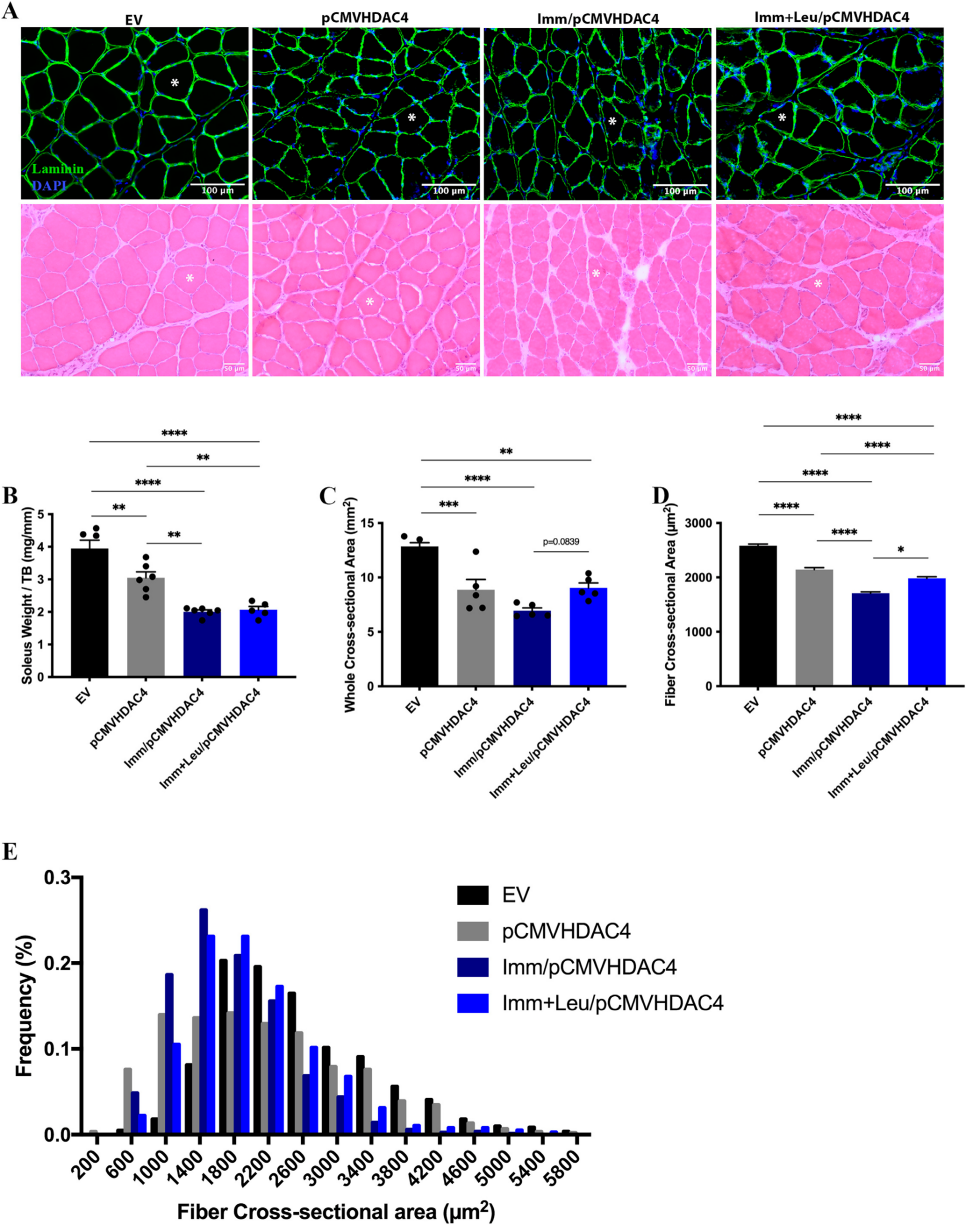
Soleus muscle morphometric analysis after 7 days of hind limb immobilization and leucine supplementation in animals that overexpressed HDAC4. (A) Representative immunofluorescence photomicrographs of soleus muscle, laminin (green) and DAPI (blue, used for nuclei identification) (scale bar 100 μm) and hematoxylin–eosin staining (scale bar 50 μm). The asterisk corresponds to the same fiber in both HE and immunofluorescence technique. (B) Soleus muscle mass, (C) whole muscle CSA, (D) fiber CSA and (E) fiber distribution from EV, pCMVHDAC4, Imm/pCMVHDAC4 and Imm + Leu/pCMVHDAC4 groups. Data are expressed as mean ± SEM. Statistical analysis included the two‐way ANOVA test followed by Tukey's *post hoc*. *p < 0.05; **p < 0.01; ***p < 0.001; ****p < 0.0001 (*n* = 5 per group).

We then challenged the leucine anti‐atrophic effect on soleus muscle that overexpressed HDAC4 by adding hindlimb immobilization (Figure 2). Analysis of the integrity and structure of soleus muscle tissue after electroporation did not reveal any significant changes, with fibers retaining their normal polygonal shape (Figure 2A). Regarding soleus mass, we observed a decrease of 23% in the pCMVHDAC4 group compared to the EV group, and this drop was even more pronounced in both immobilized groups (50% in Imm/pCMVHDAC4 and 48% in the Imm + Leu/pCMVHDAC4) compared to the EV group (Figure 2B). The soleus whole CSA decreased by 31% in the pCMVHDC4 group compared to the EV group, and again, the drop was more pronounced in the Imm/pCMVHDAC4 group (46%) compared to the EV group (Figure 2C); leucine supplementation presented a strong tendency of protection in this parameter with a lesser drop of 24% compared to the Imm/pCMVHDAC4 group (*p* = 0.0839, Figure 2C). Concerning fiber CSA, we observed a decrease of 17% in the pCMVHDC4 group, which was also pronounced in the Imm/pCMVHDAC4 group (34%) compared to the EV group; leucine supplementation showed a protection of the decrease of 14% compared to the Imm/pCMVHDAC4 group (Figure 2D). This protection was supported by a decreased frequency of smaller fibers in the Imm + Leu/pCMVHDAC4 group compared to the Imm/pCMVHDAC4 group.

### 3.3 Effect of Leucine Supplementation on HDAC4 Protein Expression in HDAC4 Overexpressed Soleus Muscle

**FIGURE 3:**
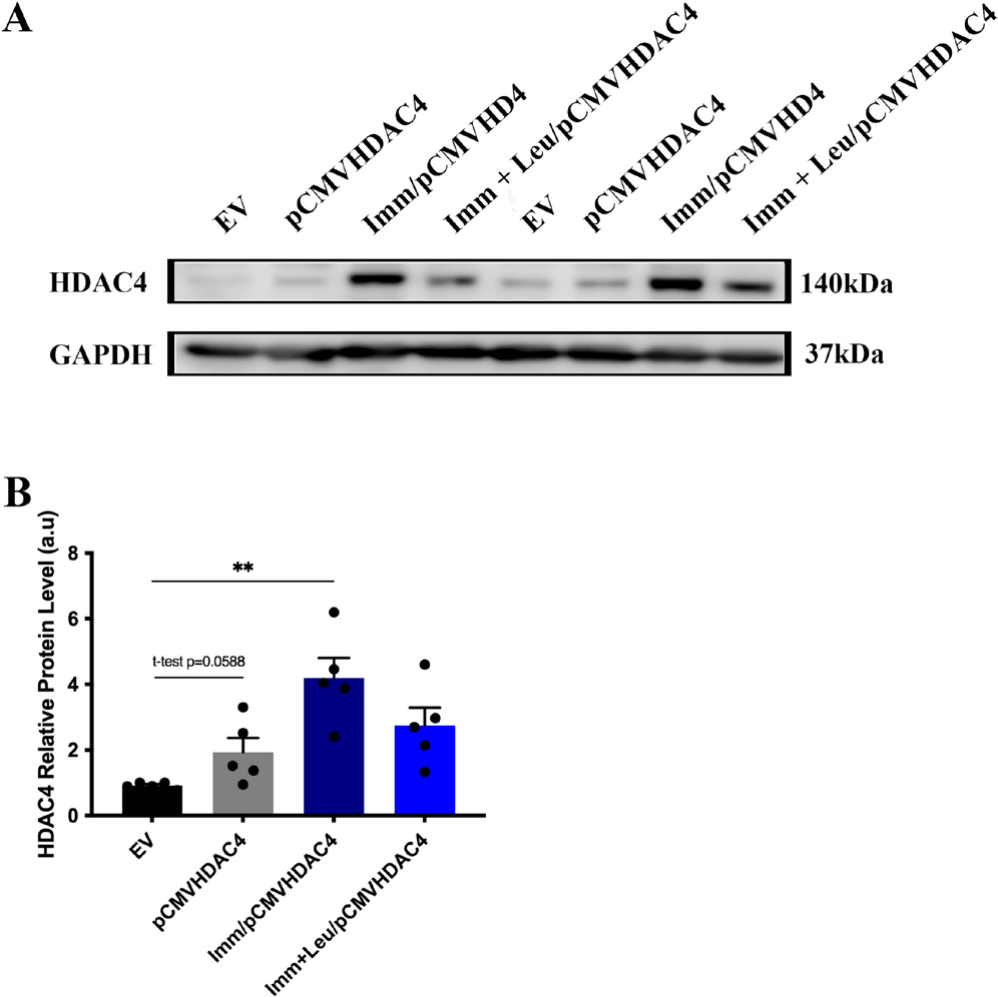
HDAC4 protein expression analysis after 7 days of hind limb immobilization and leucine supplementation in animals that overexpressed HDAC4. (A) HDAC4 and GAPDH western blot showing representative bands. (B) Densitometry analysis by using GAPDH protein level as loading control. Data are expressed as mean ± SEM. Statistical analysis included the two‐way ANOVA test followed by Tukey's *post hoc*. **p < 0.01 (*n* = 5 per group).

We observed a significant increase in HDAC4 protein expression in the Imm/pCMVHDAC4 group compared to the EV group (Figure 3B). Additionally, no statistically significant difference was observed in the pCMVHDAC4 and Imm + Leu/pCMVHDAC4 groups when compared to the control group (EV) regarding HDAC4 protein levels (Figure 3B).

### 3.4 Impact of Leucine on Gene Expression of HDAC4 Pathway‐Related Components in Overexpressed Soleus Muscle

**FIGURE 4:**
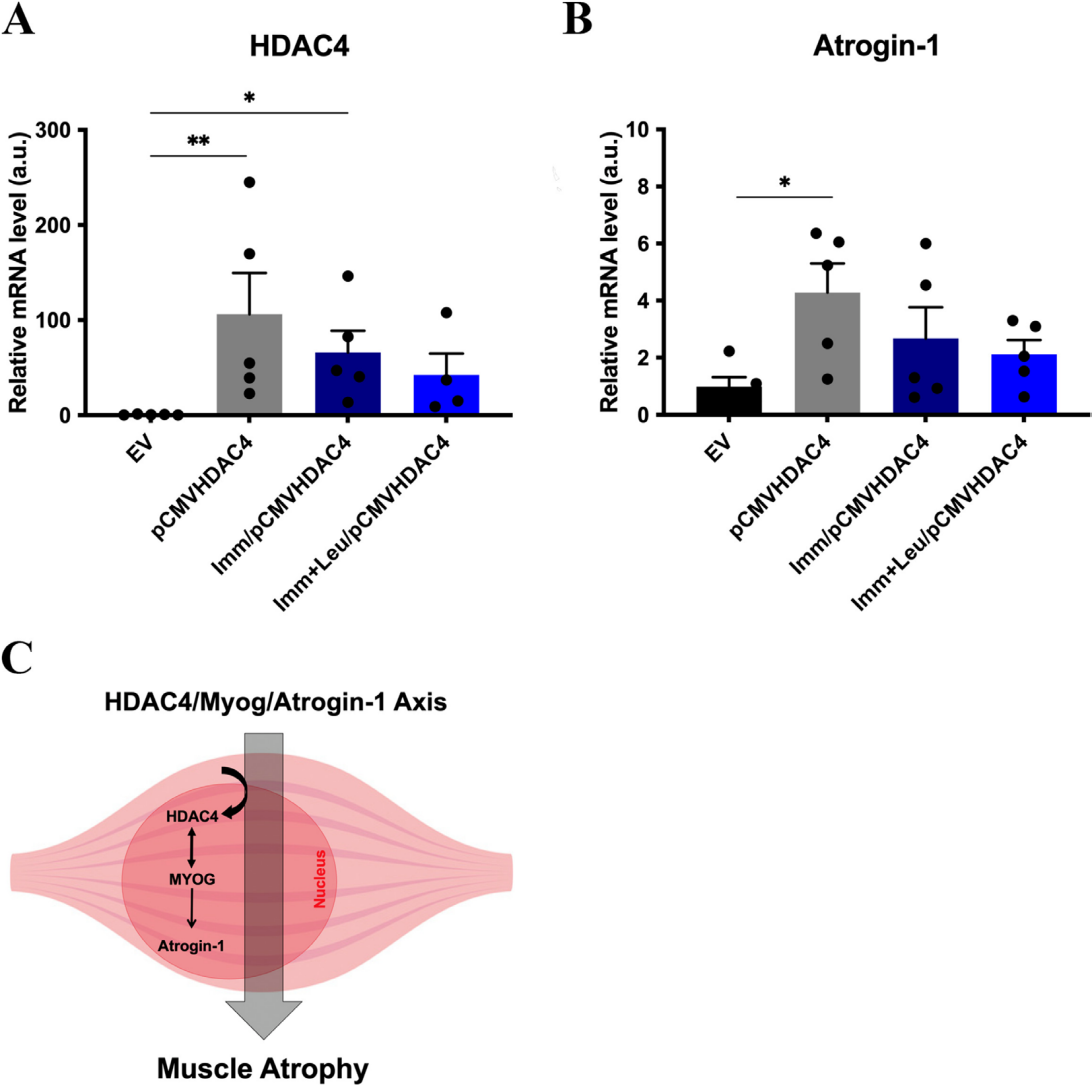
Effect of leucine on HDAC4 canonical pathway in animals that overexpressed HDAC4. (A) HDAC4 and (B) Atrogin‐1 mRNA levels were determined by qPCR. (C) Schematic illustrating HDAC4 action in the skeletal muscle fiber nucleus. Cyclophilin A expression was used as housekeeping. Data were expressed as mean ± SEM. Statistical analysis included one‐way ANOVA followed by Tukey's *post hoc* test. *p < 0.05; **p < 0.01 (*n* = 5 per group).

We investigated molecular components related to the atrophic effect of HDAC4, specifically Atrogin‐1/MAFbx gene expression (Figure 4). HDAC4 mRNA expression was increased in the pCMVHDAC4 and Imm/pCMVHDAC4 groups compared to the EV group (Figure 4A). Interestingly, no statistically significant difference was observed in the Imm + Leu/pCMVHDAC4 group when compared across all groups (Figure 4A).

Following the HDAC4 atrophic axis, we observed an increase in Atrogin‐1/MAFbx mRNA expression in the pCMVHDAC4 group compared to the EV group (Figure 4B).

### 3.5 Leucine Reduces the Percentage of HDAC4 Positive Nuclei Accumulation in Overexpressed Soleus Muscle

**FIGURE 5:**
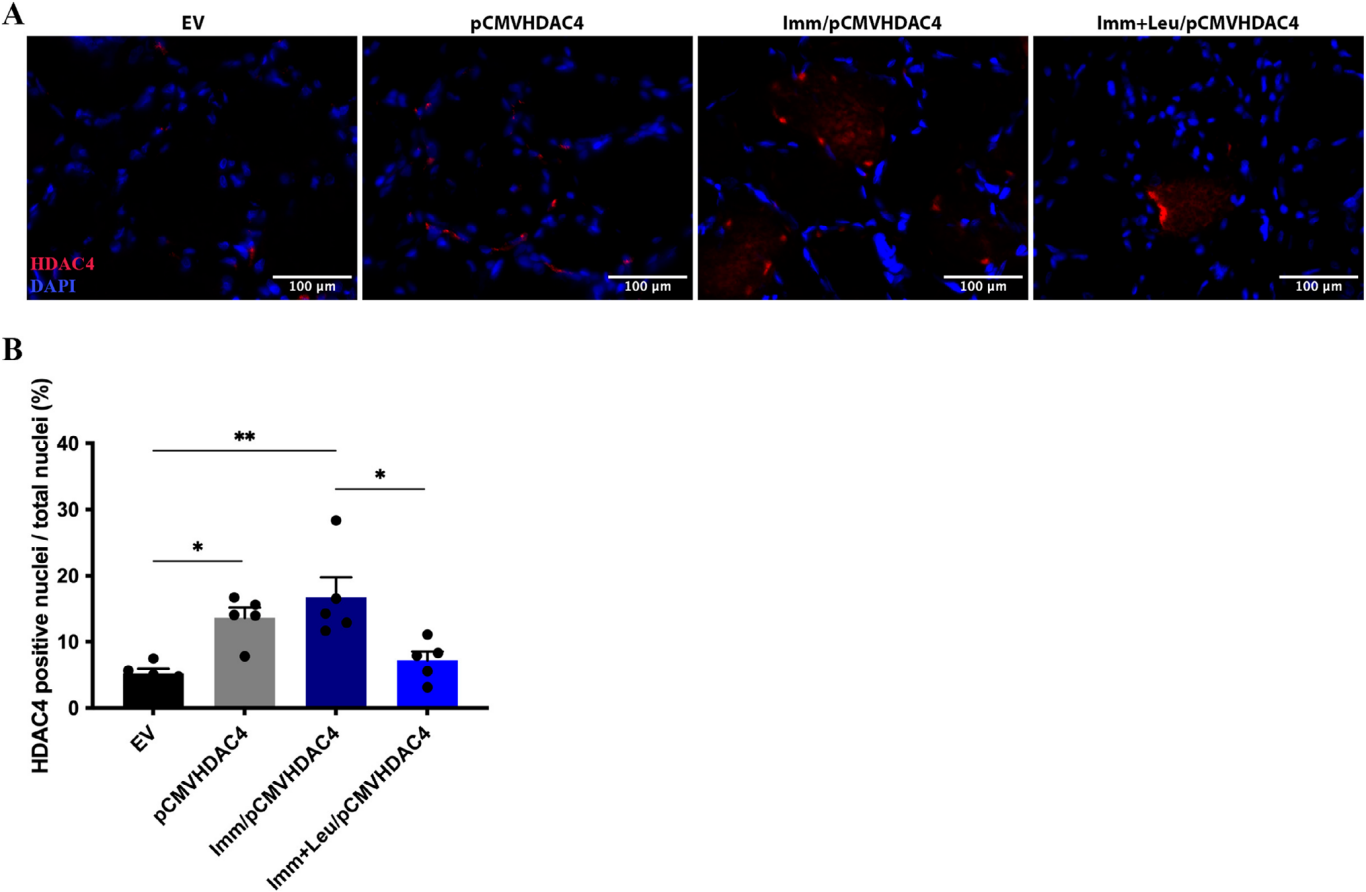
HDAC4 nuclei localization after 7 days of hind limb immobilization and leucine supplementation in animals that overexpressed HDAC4. (A) Representative immunofluorescence photomicrographs of HDAC4 in soleus muscle, HDAC4 (red), DAPI (blue, used for nuclei identification) (scale bar 100 μm). (B) Number of positive HDAC4 nuclei per total nuclei (%). Data were expressed as mean ± SEM. Statistical analysis included two‐way ANOVA followed by Tukey's *post hoc* test. *p < 0.05; **p < 0.01 (*n* = 5 per group).

We observed that the overexpression of HDAC4 induced an increase in the number of HDAC4‐positive nuclei (approximately 3‐fold) in the pCMVHDAC4 group, an effect that persisted in the Imm/pCMVHDAC4 group when compared to the EV group (Figure 5B). Leucine supplementation significantly attenuated this effect (approximately 57% less positive nuclei) when comparing the Imm + Leu/pCMVHDAC4 group to the Imm/pCMVHDAC4 group (Figure 5B). In order to investigate whether HDAC4‐positive cells are associated with muscle fiber or satellite cell nuclei, we analyzed Pax7‐positive nuclei and found no significant variation, indicating that satellite cells are not significantly involved (Figure S5).

## 4 Discussion

In this study, we showed that HDAC4 overexpression induces skeletal muscle atrophy, leading to a loss in soleus mass, whole muscle CSA, and fiber CSA. Notably, even under high levels of HDAC4, leucine still maintained protection against skeletal muscle atrophy, preserving fiber CSA. This protective effect was accompanied by reduced HDAC4‐positive nuclei localization in immobilized rats overexpressing HDAC4. Here we confirmed that leucine supplementation effectively mitigated decreases in CSA and force (Figure 1), consistent with previous studies [6, 15, 19]. Interestingly, the specific force remained unchanged across groups, suggesting that the effects of leucine are not related to intrinsic muscle contractile characteristics, but rather to muscle mass and fiber size [20].

The vast majority of studies addressing the anti‐atrophic effect of leucine are based on cast‐immobilization [21]. Nonetheless, other studies have also employed unloading and dexamethasone as methods to induce atrophy. Importantly, regardless of the model used, leucine has been consistently recognized as a potent nutritional intervention against muscle atrophy.

An important point to consider, related to muscle mass, is the similarity of the impact of HDAC4 overexpression on muscle atrophy compared to the hindlimb immobilization model (for details please see Figure 1 and Figure S3), indicating that HDAC4 overexpression alone can induce similar atrophy to 7 days of muscle inactivity, as both interventions contribute similarly to muscle atrophy. Additional support for this interpretation arises from the combination of both interventions, where HDAC4 overexpression combined with hindlimb immobilization resulted in a two‐fold increase in effect compared to HDAC4 overexpression alone (Figure 2).

Regarding the molecular effects of HDAC4 overexpression, we observed an increase in both HDAC4 protein and mRNA levels in the HDAC4 overexpression groups. Interestingly, leucine supplementation showed a potential to suppress HDAC4 protein and mRNA levels (Figures 3 and 4). Accordingly, in the pCMVHDAC4 group, Atrogin‐1 mRNA levels were elevated compared to the EV group, validating the known link between HDAC4 and the ubiquitin‐proteasome system [4].

Since there are no existing studies specifically overexpressing HDAC4 in skeletal muscle for direct comparison, we refer to a study where the effects of Myogenin (a downstream target of HDAC4) overexpression on muscle fiber CSA and expression of the E3 ubiquitin ligases MuRF‐1 and Atrogin‐1 were addressed [4]. The authors found that overexpression of myogenin induced muscle atrophy, reducing TA fiber CSA by approximately 40% [4]; interestingly, in line with our results, confirming both the potential to induce muscle atrophy and the positive link between the HDAC4 and Myogenin axis. In addition, the authors demonstrated upregulation of MuRF1 and atrogin‐1 expression, which is consistent with our findings of increased Atrogin‐1 mRNA levels in the pCMVHDAC4 group (Figure 4C) [4].

An important aspect of this study concerns the potential cellular mechanisms by which leucine exerts its effects. Since nuclear localization of HDAC4 is required to induce transcription of atrophy‐related genes such as Atrogin‐1, we examined the effect of leucine on HDAC4 nuclear localization. Previously, we demonstrated that leucine reduced HDAC4‐positive nuclear localization by approximately 50% in hindlimb immobilized animals [6]. In this study, we observed that even with forced overexpression of HDAC4, leucine is still able to mitigate the access of this protein to the nucleus, reducing HDAC4‐positive nuclear localization (Figure 5). In addition, we found an increase in HDAC4‐positive nuclear localization in both the pCMVHDAC4 and pCMVHDAC4/Imm groups, by 2‐fold and 4‐fold, respectively (Figure 5). These results on HDAC4 nuclear localization are in line with the increase in HDAC4 protein levels detected by western blotting (Figure 5). As discussed above, the correlation between protein levels and nuclear localization suggests that the nuclear localization of HDAC4 reflects the fraction of functioning protein.

Another key aspect regarding the potent effect we observed herein involves the understanding of the mechanisms by which leucine can keep HDAC4 outside the nucleus, and post‐translational modifications are potential factors. Protein phosphorylation, one of the most extensively studied and well‐understood modifications, is known to regulate protein function, activity, and stability by targeting specific amino acid residues via protein kinases [22]. Specifically, phosphorylation of certain amino acids in HDAC4 can facilitate the translocation of this protein from the nucleus to the cytoplasm [23, 24]. HDAC4 can be phosphorylated by the calcium/calmodulin‐dependent protein kinases (CaMK) and protein kinase A (PKA). Phosphorylation of HDAC4 at S246, S467, and S632 by CaMKII enhances nuclear export and prevents the nuclear import of HDAC4, resulting in down‐regulation of HDAC4 target genes [25]. Our previous studies demonstrated that leucine upregulates CAMKII mRNA levels during hindlimb immobilization [6], indicating a potential mechanism by which leucine could influence the induction of HDAC4 phosphorylation at Ser632 (Figure S4) and its decreased nuclear localization (Figure 5). In addition, we have recently shown that leucine increases PKA protein levels in cardiomyocytes from HFpEF rats [14]. Active PKA in the nucleus can phosphorylate HDAC4 at Ser‐740, leading to its nuclear export [26]. However, other studies have reported that PKA promotes nuclear accumulation of HDAC4 [27, 28], suggesting that the effect of PKA on HDAC4 localization depends on the specific phosphorylation site. Further research investigating the role of leucine in the control of nuclear protein transport would be crucial to elucidate the mechanisms by which leucine influences this process.

Given the potential of leucine as a translational therapeutic approach, it is important to compare its effects in rodents and humans. A recent review showed that the most significant benefits of leucine supplementation are the increase in muscle CSA and strength [29]. Another study by English et al. supplemented humans with leucine and found that it attenuated lean mass loss after 7 days of bed rest [30]. However, a study by Edwards et al. provided high‐dose leucine supplementation to young, healthy individuals and found no significant attenuation of functional losses associated with 7 days of limb immobilization [31]. It is important to highlight that the variability in skeletal muscle improvement may be influenced by factors such as age, sex, dosage, and postprandial uptake [30, 31, 32, 33, 34, 35].

## 5 Limitations

First, the relatively large variation among samples in certain groups narrowed the statistical analysis, despite clear directional effects. Second, all experiments were performed in male rats, limiting the generalizability of the results to this gender. Also, only one model (cast‐immobilization) of skeletal mass loss was utilized. Future studies using other models such as denervation, tail suspension, and dexamethasone treatment will clarify how broad the anti‐atrophic effect of leucine is. Third, we did not analyze all proteins involved in the HDAC4 pathway, such as myogenin and Atrogin‐1.

## 6 Conclusions

In summary, the results herein suggest that HDAC4 overexpression plays a major role in inducing skeletal muscle mass loss during hindlimb immobilization, and the leucine anti‐atrophic effect strongly mitigates nuclear translocation of HDAC4. Further studies employing HDAC4 KO models and leucine supplementation would be valuable to understand the specific mechanisms behind this protection and whether it is related to hindlimb immobilization or HDAC4 suppression. Moreover, we acknowledge the need for additional studies to enhance the translational relevance of findings from rodent models to human contexts.

## Author Contributions

**Paula K. N. Alves:** conceptualization, investigation, writing – original draft, methodology, validation, visualization, formal analysis. **André Cruz:** methodology. **William J. Silva:** methodology. **Afonso M. Melazzo:** methodology. **Siegfried Labeit:** writing – review and editing, resources. **Volker Adams:** writing – review and editing. **Anselmo S. Moriscot:** conceptualization, funding acquisition, writing – review and editing, formal analysis, resources, supervision, data curation, project administration.

## Ethics Statement

We confirm that we have read the Journal's position on issues involved in ethical publication and affirm that this report is consistent with those guidelines.

## Conflicts of Interest

The authors declare no conflicts of interest.

## Supporting information


**Figure S1.** Experimental design for all experiments performed. A total of 64 male Wistar rats (~280 g) was used in this study and divided into 3 independent experiments: (A) The animals were randomized into four groups: Control group (Control), Leucine group (Leu), Immobilized group (Imm 7d) and Immobilized + Leucine group (Imm 7d + Leu). After 7 days of hind limb immobilization, the soleus muscle was harvested for histological and molecular analyses. (B) The animals were randomized into four groups: EV 4d group, pCMVHDAC4 4d group, EV 8d group and pCMVHDAC4 8d group. Four and eight days after electrotransfer the soleus muscle was harvested for histological and molecular analyses. (C) One day after eletrotransfer, the animals were randomized into four groups: EV group, pCMVHDAC4 group, Imm/pCMVHDAC4 and Imm + Leu/pCMVHDAC4. After 11 days of electrotransfer and 7 days of hind limb immobilization, the soleus muscle was harvested for histological and molecular analyses. (D) Symbols legend.

**Figure S2.** Plasmid information and eletrotansfer efficiency. (A) Plasmid map. (B) Primer’s hybridization sites and (C) Sequences. (D) Representative western blot bands of DDK after 4 and (E) 8 days of eletrotransfer. (F) Densitometric analysis of DDK (plasmid flag) after 4 and 8 days of eletrotransfer. Data are expressed as mean ± SD. Statistical analysis included the unpaired *t*‐student test. *p < 0.05 verusus corresponding EV (*n* = 3–4 per group).

**Figure S3.** Tissue characterization of rat’s soleus muscle after HDAC4 overexpression for 4 and 8 days. (A) Soleus muscle wet weight after 4 and (B) 8 days of eletrotransfer. (C) Soleus muscle average of whole muscle CSA after 4 and (D) 8 days eletrotransfer. (E) Soleus muscle average of fiber CSA after 4 and (F) 8 days of eletrotransfer. (G) Fiber distribution from EV and pCMVHDAC4 after 4 and (H) 8 days of eletrotransfer. (I) Representative photomicrographs of soleus muscle fiber stained with hematoxylin and eosin from groups EV (animals electroporated with a plasmid containing an empty vector) and pCMVHDAC4 (animals electroporated with a plasmid containing HDAC4 mRNA). (J) Representative western blot bands of HDAC4 after 4 and 8 days of eletrotransfer and (K) densitometric analysis of HDAC4 after 4 days and 8 days of eletrotransfer. Data are expressed as mean ± SD. Statistical analysis included the unpaired *t*‐student test. *p < 0.05 and ****p < 0.0001 vs. corresponding EV (*n* = 3–4 per group).

**Figure S4.** Analysis of leucine supplementation on expression of HDAC4pSer632 after 7 days of hind limb immobilization. (A) Western blot representative bands of HDAC4, HDAC4p Ser632, and GAPDH. (B) Densitometric analysis in control, immobilized and immobilized supplemented with leucine after 7 days. Data are expressed as mean ± SEM. Statistical analysis included the one‐way anova test followed by Dunn’s post hoc. ***p < 0.001 versus control group (*n* = 7 per group).

**Figure S5.** Pax7 nuclei localization after 7 days of hind limb immobilization and leucine supplementation. (A) Representative immunofluorescence photomicrographs of Pax7 immunolabeling (red) in soleus muscle and DAPI (blue, used for nuclei identification) (scale bar 50 μm). (B) Bar histogram representing the percentage of positive Pax7 nuclei (%). Data were expressed as mean ± SEM. Statistical analysis included two‐way anova followed by Tukey’s post hoc test (*n* = 5 per group).

**Figure S6.** Figures information.

**Figure S7.** Figures information.

**Data S1.** Supporting Information.

**Table S1.** List of genes and primers used for gene expression analysis.

## Data Availability

The data that support the findings of this study are available from the corresponding author upon reasonable request.
