# Supplementary figures and images for: Leucine Supplementation Counteracts the Atrophic Effects of HDAC4 in Rat Skeletal Muscle Submitted to Hindlimb Immobilization
Source: Muscle Nerve. 2025 Apr 4;72(1):139–48. doi: 10.1002/mus.28411 (PMC12138493; doi:10.1002/mus.28411)

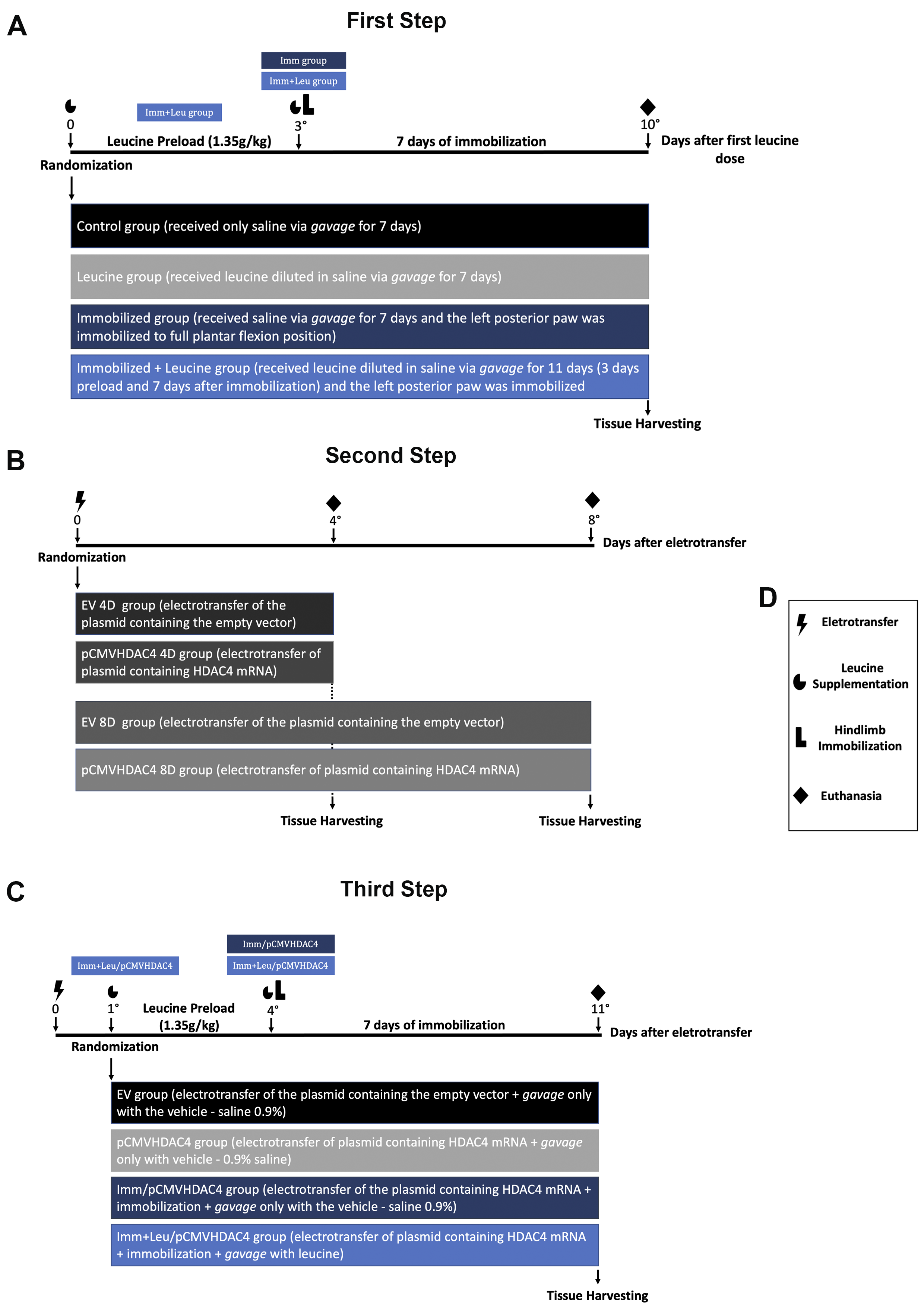

Supplement: Supplementary file 1 — Figure S1. Experimental design for all experiments performed. A total of 64 male Wistar rats (~280 g) was used in this study and divided into 3 independent experiments: (A) The animals were randomized into four groups: Control group (Control), Leucine group (Leu), Immobilized group (Imm 7d) and Immobilized + Leucine group (Imm 7d + Leu). After 7 days of hind limb immobilization, the soleus muscle was harvested for histological and molecular analyses. (B) The animals were randomized into four groups: EV 4d group, pCMVHDAC4 4d group, EV 8d group and pCMVHDAC4 8d group. Four and eight days after electrotransfer the soleus muscle was harvested for histological and molecular analyses. (C) One day after eletrotransfer, the animals were randomized into four groups: EV group, pCMVHDAC4 group, Imm/pCMVHDAC4 and Imm + Leu/pCMVHDAC4. After 11 days of electrotransfer and 7 days of hind limb immobilization, the soleus muscle was harvested for histological and molecular analyses. (D) Symbols legend. [file MUS-72-139-s006.tif]

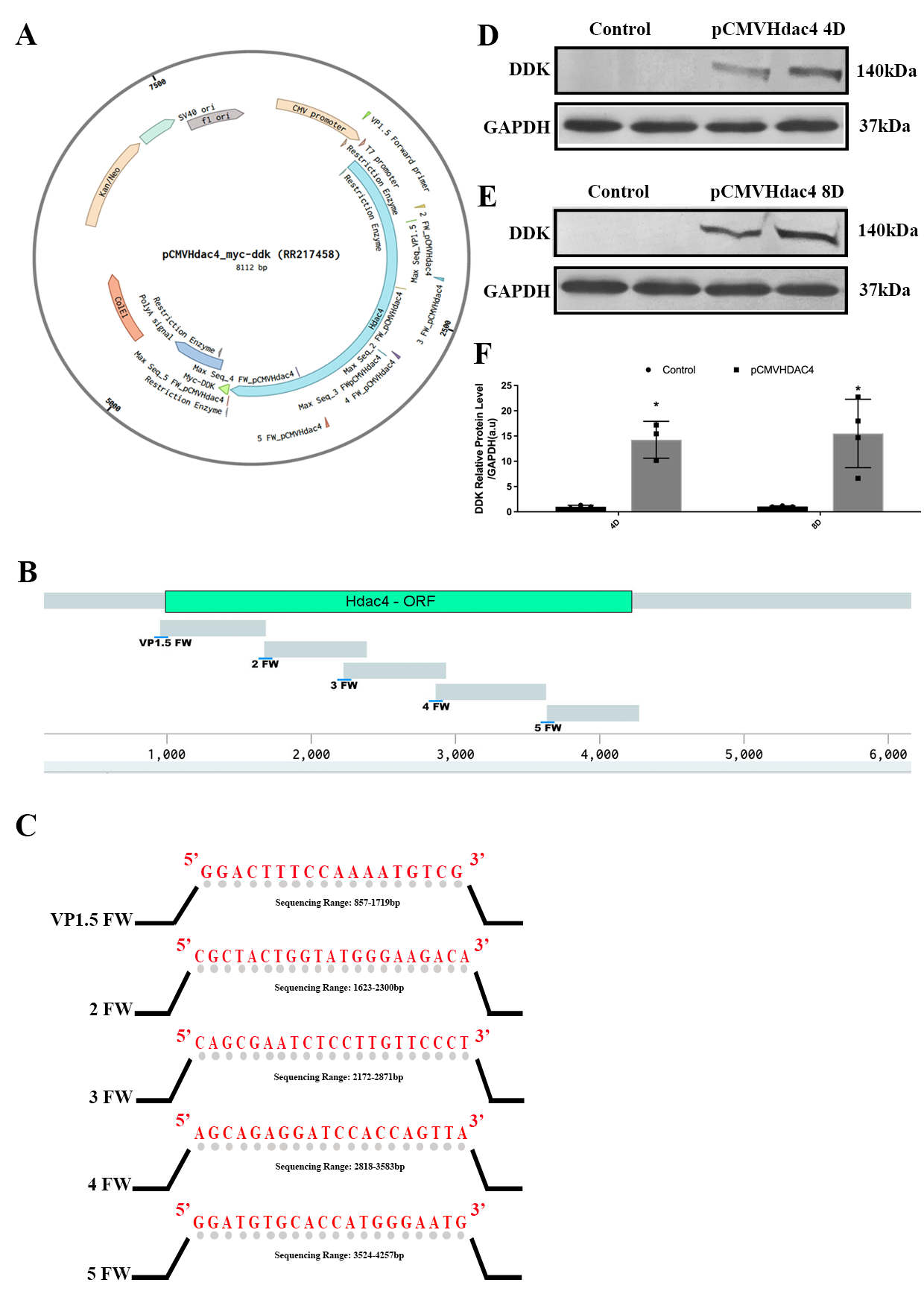

Supplement: Supplementary file 2 — Figure S2. Plasmid information and eletrotansfer efficiency. (A) Plasmid map. (B) Primer’s hybridization sites and (C) Sequences. (D) Representative western blot bands of DDK after 4 and (E) 8 days of eletrotransfer. (F) Densitometric analysis of DDK (plasmid flag) after 4 and 8 days of eletrotransfer. Data are expressed as mean ± SD. Statistical analysis included the unpaired t‐student test. *p < 0.05 verusus corresponding EV (n = 3–4 per group). [file MUS-72-139-s005.tif]

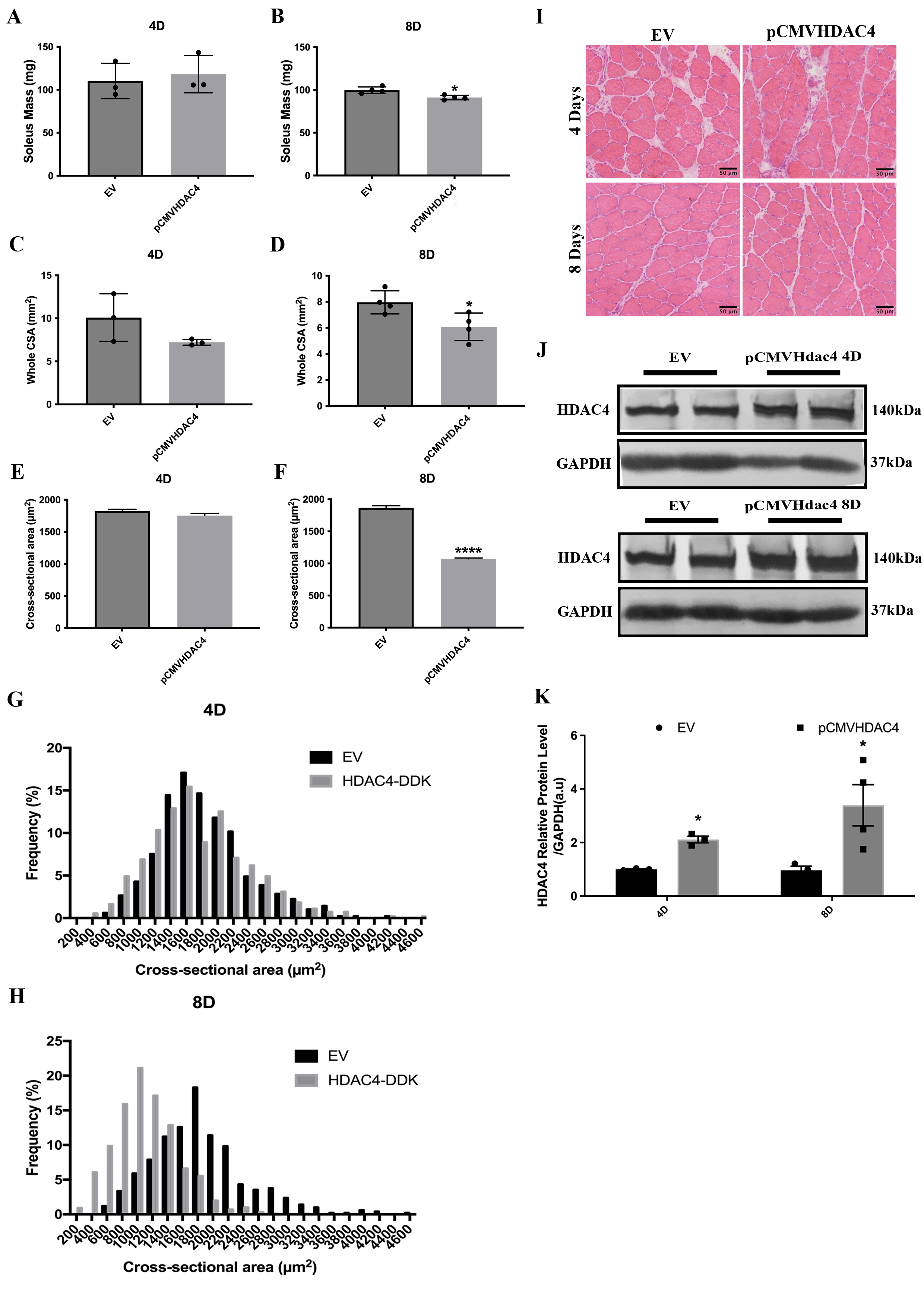

Supplement: Supplementary file 3 — Figure S3. Tissue characterization of rat’s soleus muscle after HDAC4 overexpression for 4 and 8 days. (A) Soleus muscle wet weight after 4 and (B) 8 days of eletrotransfer. (C) Soleus muscle average of whole muscle CSA after 4 and (D) 8 days eletrotransfer. (E) Soleus muscle average of fiber CSA after 4 and (F) 8 days of eletrotransfer. (G) Fiber distribution from EV and pCMVHDAC4 after 4 and (H) 8 days of eletrotransfer. (I) Representative photomicrographs of soleus muscle fiber stained with hematoxylin and eosin from groups EV (animals electroporated with a plasmid containing an empty vector) and pCMVHDAC4 (animals electroporated with a plasmid containing HDAC4 mRNA). (J) Representative western blot bands of HDAC4 after 4 and 8 days of eletrotransfer and (K) densitometric analysis of HDAC4 after 4 days and 8 days of eletrotransfer. Data are expressed as mean ± SD. Statistical analysis included the unpaired t‐student test. *p < 0.05 and ****p < 0.0001 vs. corresponding EV (n = 3–4 per group). [file MUS-72-139-s007.tif]

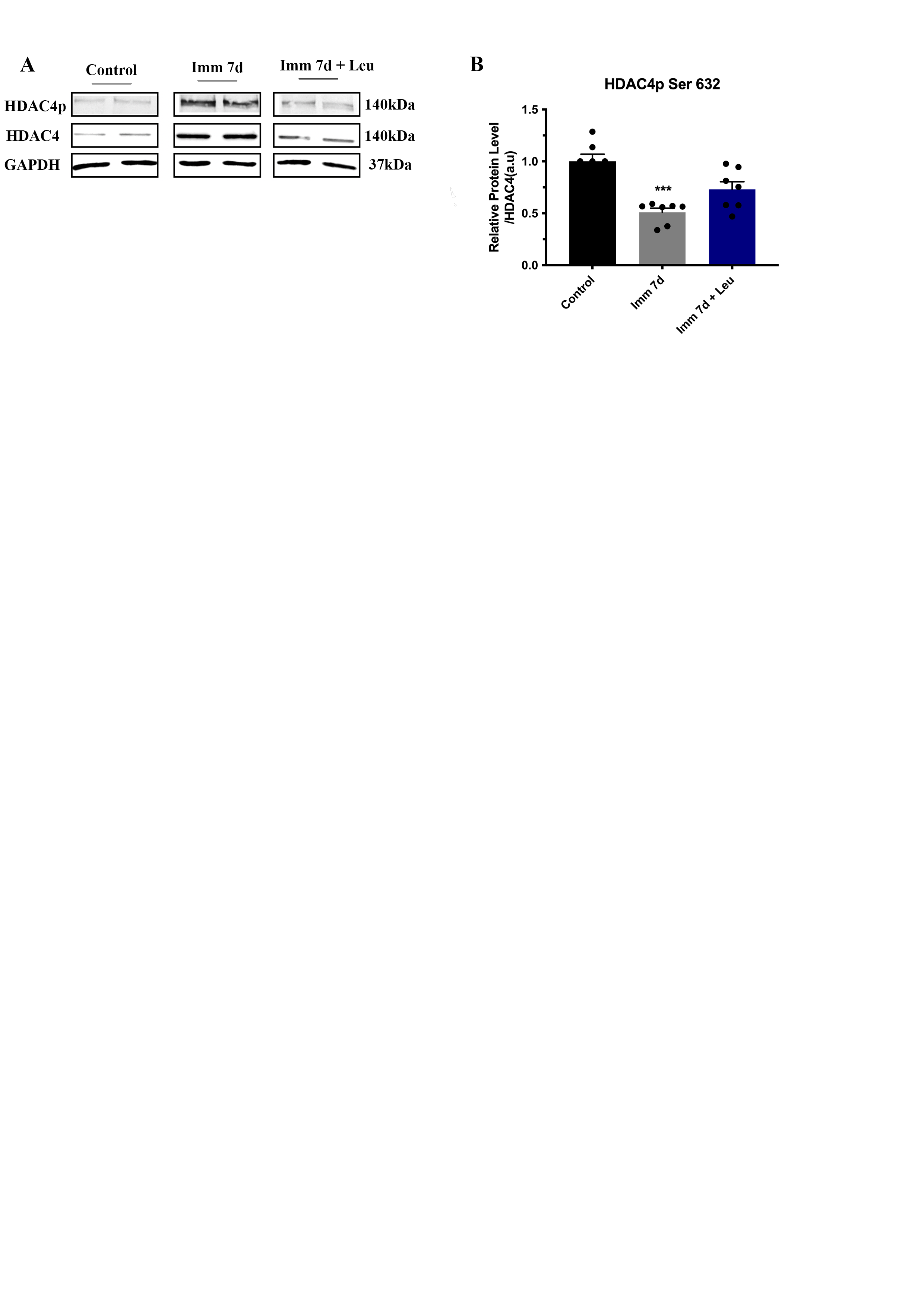

Supplement: Supplementary file 4 — Figure S4. Analysis of leucine supplementation on expression of HDAC4pSer632 after 7 days of hind limb immobilization. (A) Western blot representative bands of HDAC4, HDAC4p Ser632, and GAPDH. (B) Densitometric analysis in control, immobilized and immobilized supplemented with leucine after 7 days. Data are expressed as mean ± SEM. Statistical analysis included the one‐way anova test followed by Dunn’s post hoc. ***p < 0.001 versus control group (n = 7 per group). [file MUS-72-139-s003.tif]

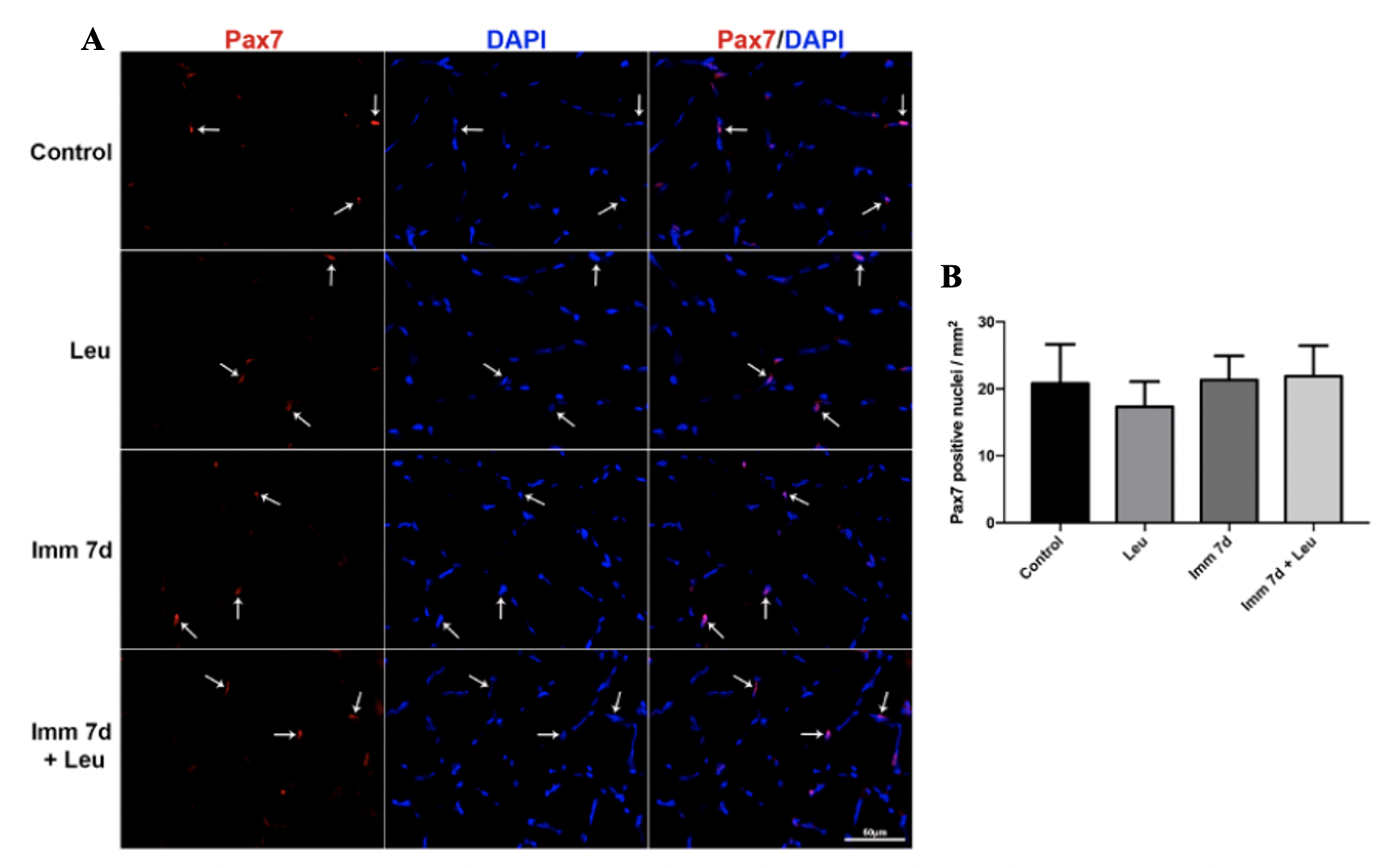

Supplement: Supplementary file 5 — Figure S5. Pax7 nuclei localization after 7 days of hind limb immobilization and leucine supplementation. (A) Representative immunofluorescence photomicrographs of Pax7 immunolabeling (red) in soleus muscle and DAPI (blue, used for nuclei identification) (scale bar 50 μm). (B) Bar histogram representing the percentage of positive Pax7 nuclei (%). Data were expressed as mean ± SEM. Statistical analysis included two‐way anova followed by Tukey’s post hoc test (n = 5 per group). [file MUS-72-139-s009.tif]

# Uncropped Blots for the Main Figures

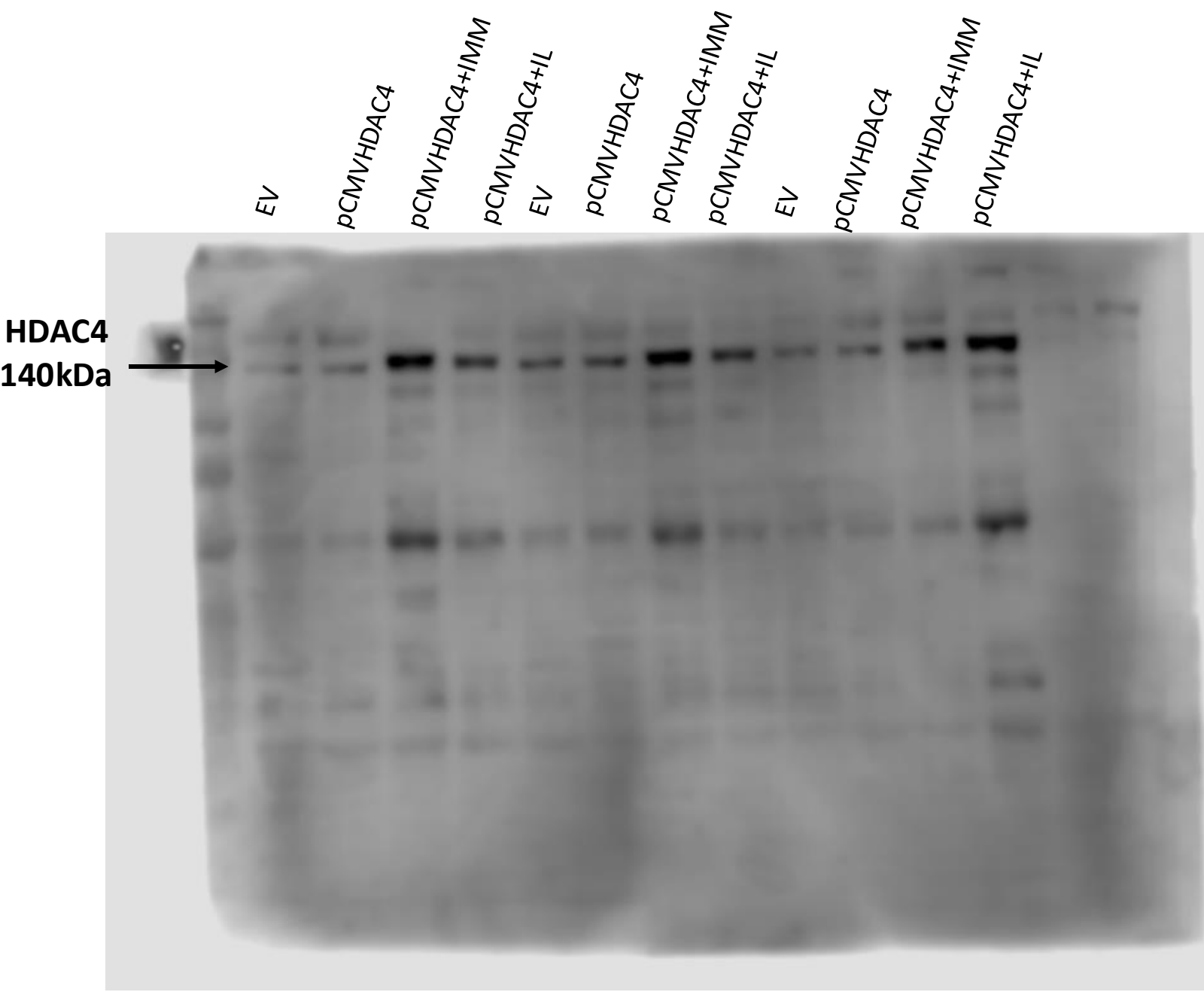

GAPDH – Incubated after HDAC4

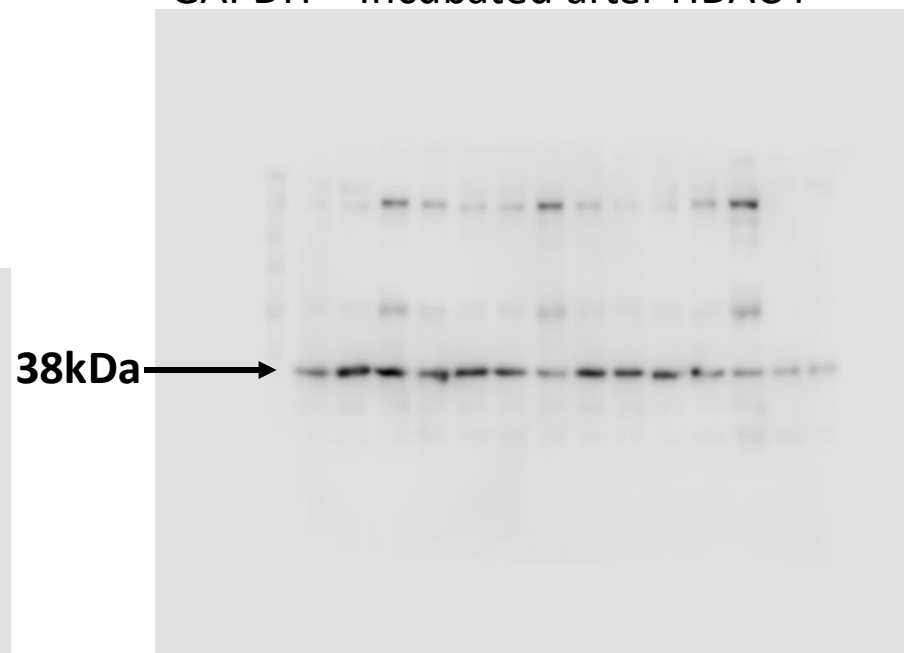

Ponceau

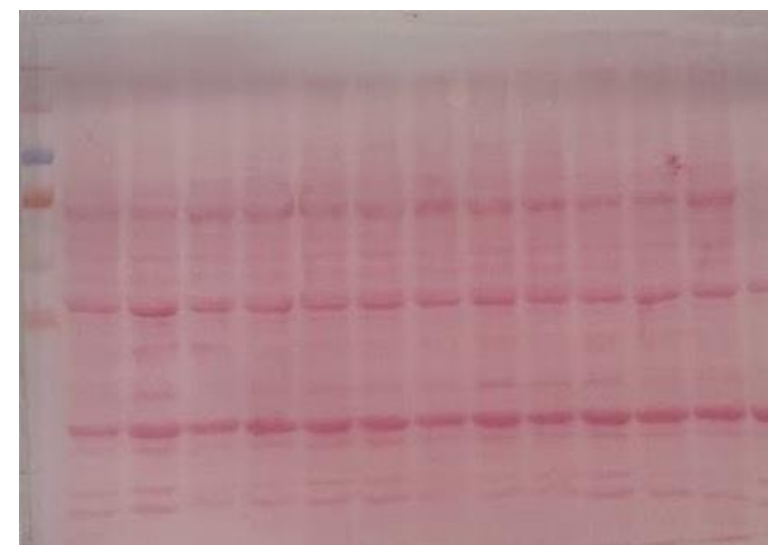

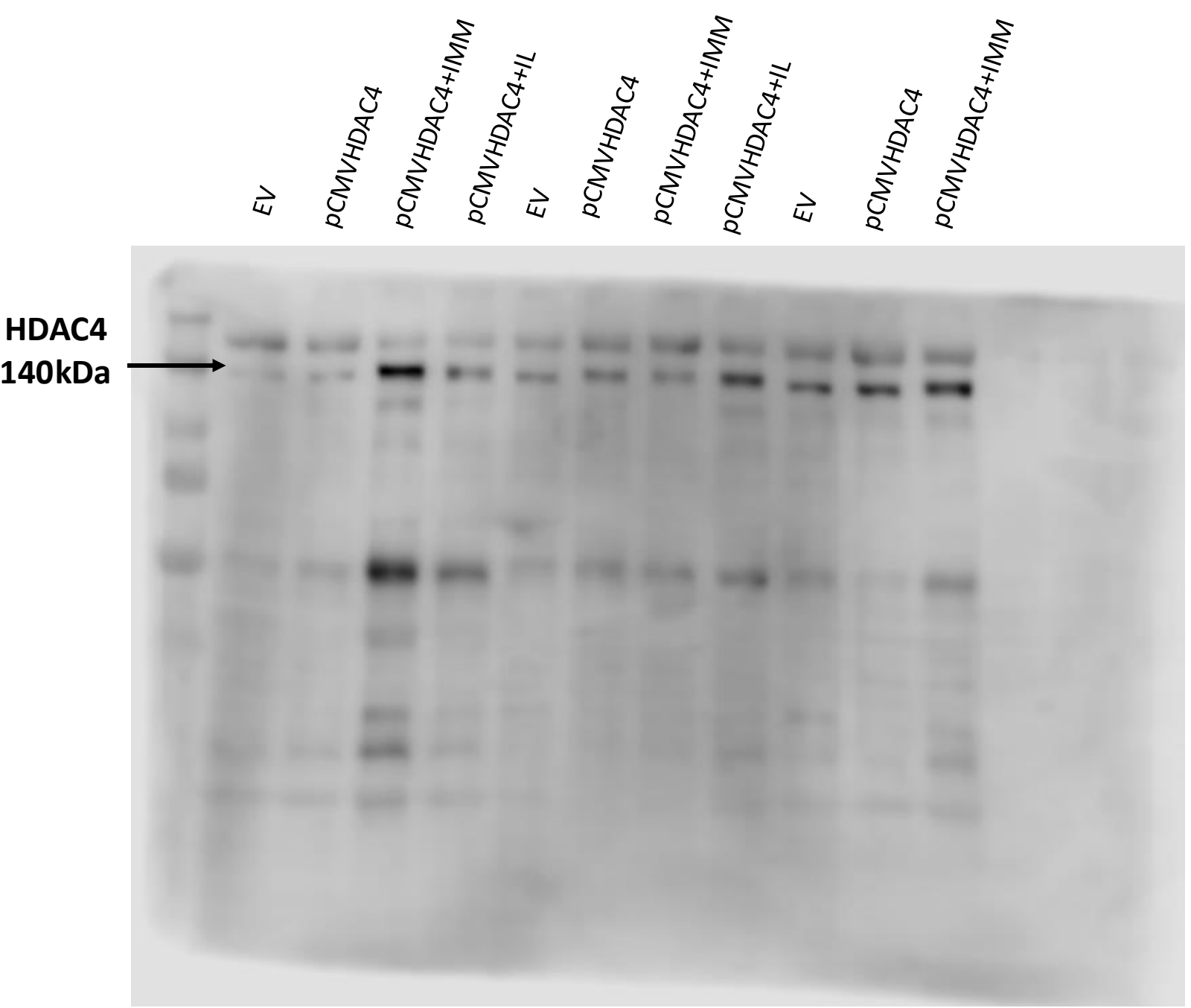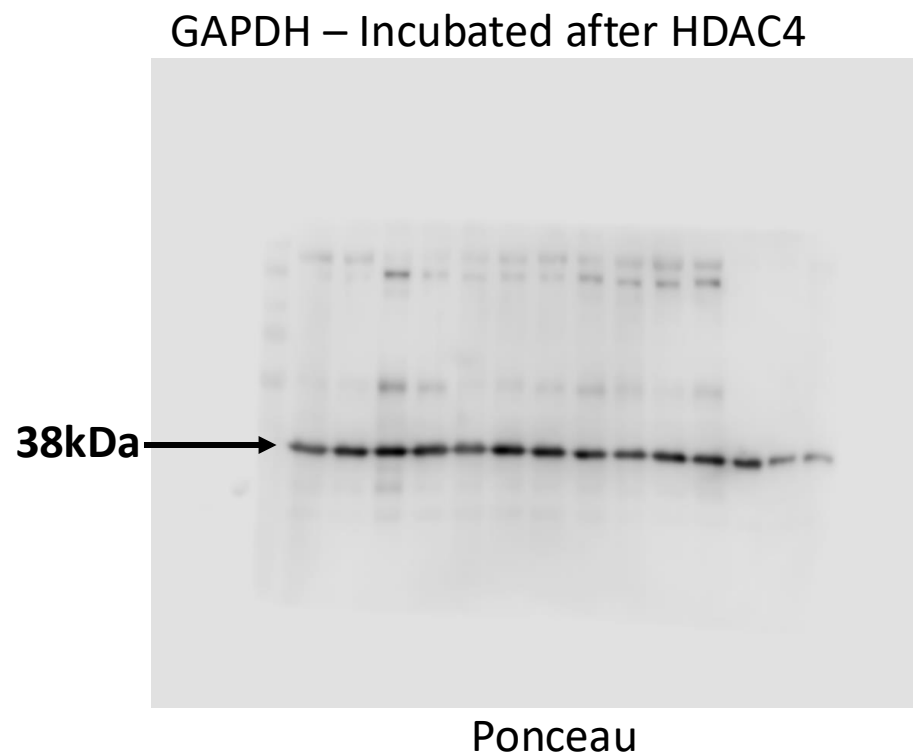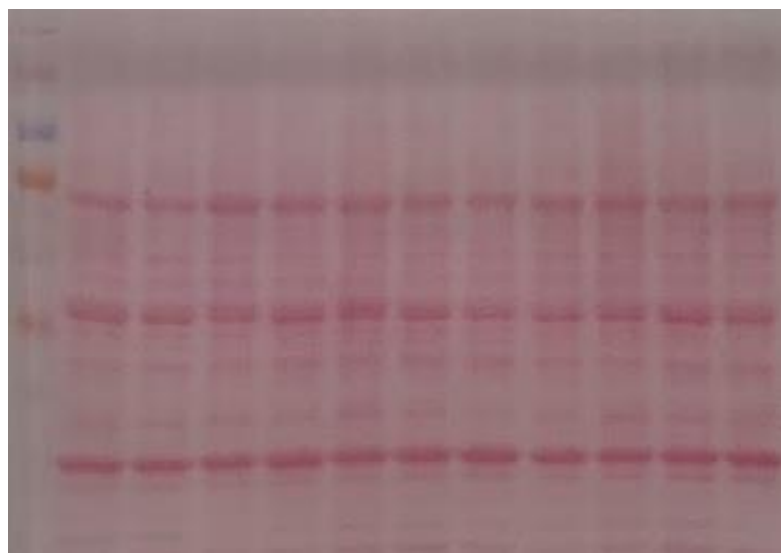

Supplement: Supplementary file 7 — Figure S7. Figures information. [file MUS-72-139-s008.pdf]
