# Supplementary material for: Leucine Supplementation Counteracts the Atrophic Effects of HDAC4 in Rat Skeletal Muscle Submitted to Hindlimb Immobilization
Source: Muscle Nerve. 2025 Apr 4;72(1):139–48. doi: 10.1002/mus.28411 (PMC12138493; doi:10.1002/mus.28411)

Uncropped Blots for the Sup Figures

EV pCMVHDAC4_4D

DDK
140kDa

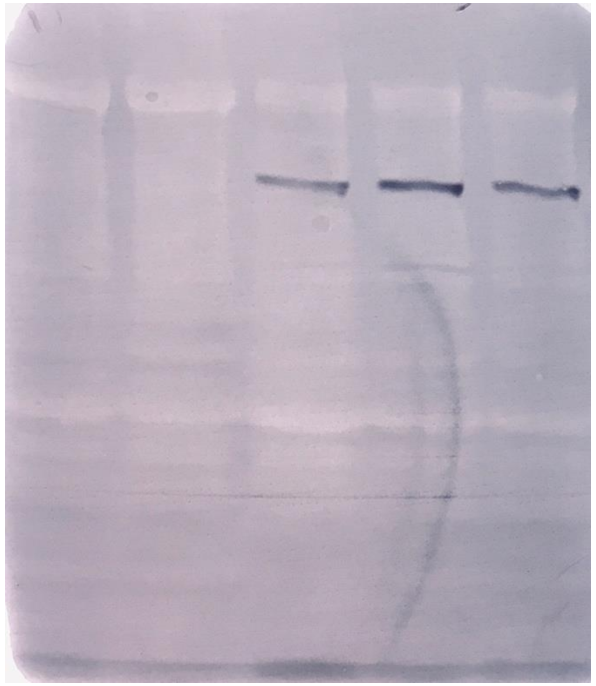

Ponceau

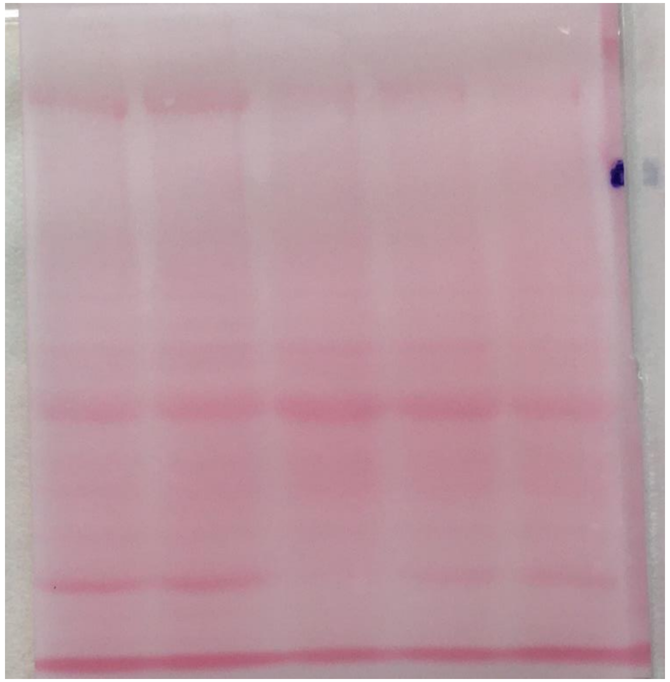

EV pCMVHDAC4_4D

HDAC4
140kDa

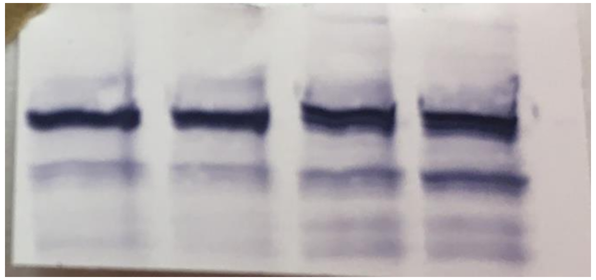

GAPDH
38kDa

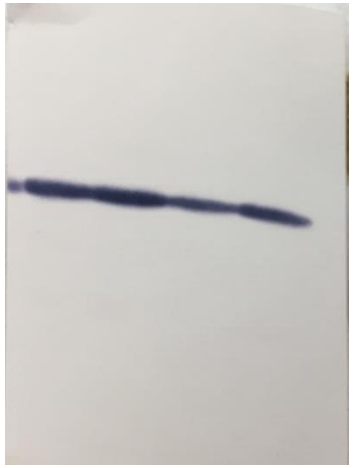

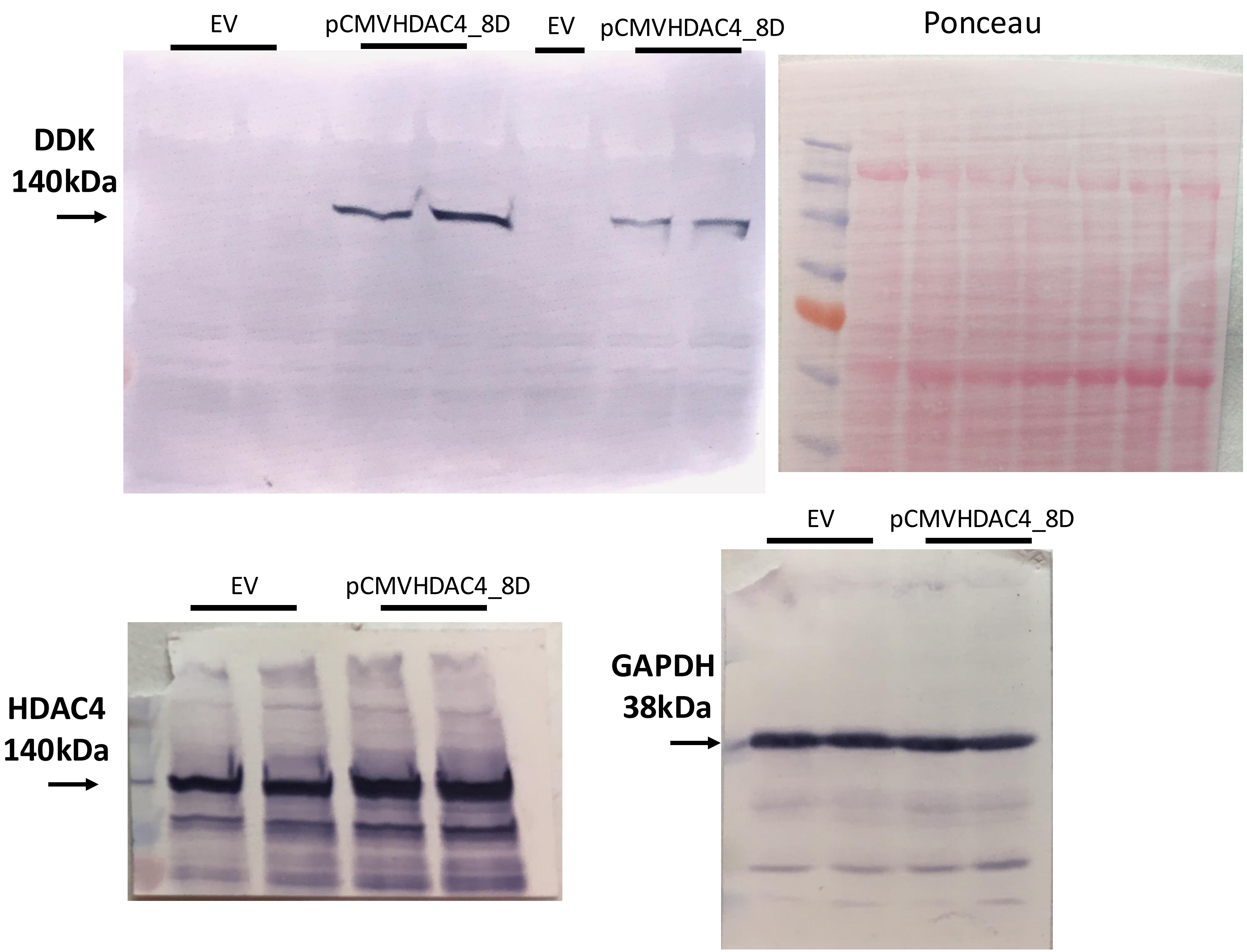
EV
pCMVHDAC4_8D
EV
pCMVHDAC4_8D
Ponceau
DDK
140kDa
EV
pCMVHDAC4_8D
HDAC4
140kDa
EV
pCMVHDAC4_8D
GAPDH
38kDa

Control Control Imm 7d Imm 7d + Leu

HDAC4p
140kDa

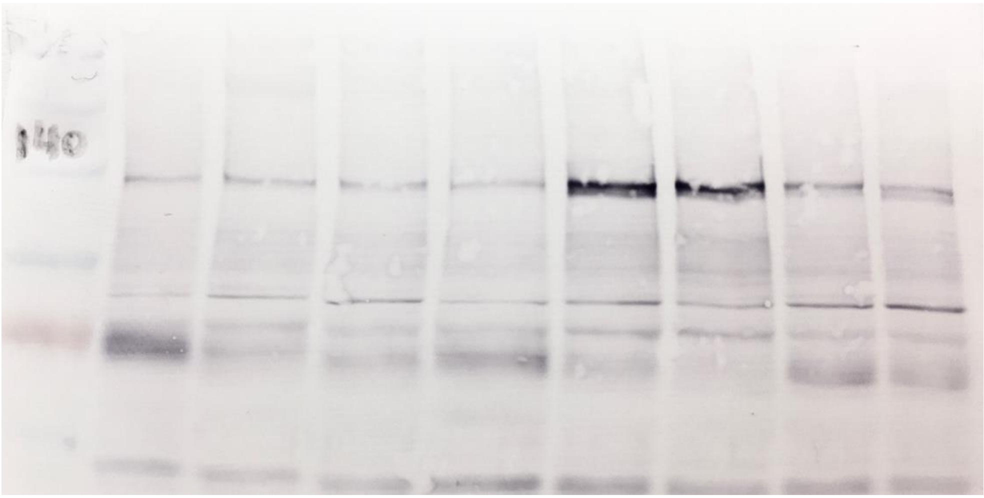

Ponceau

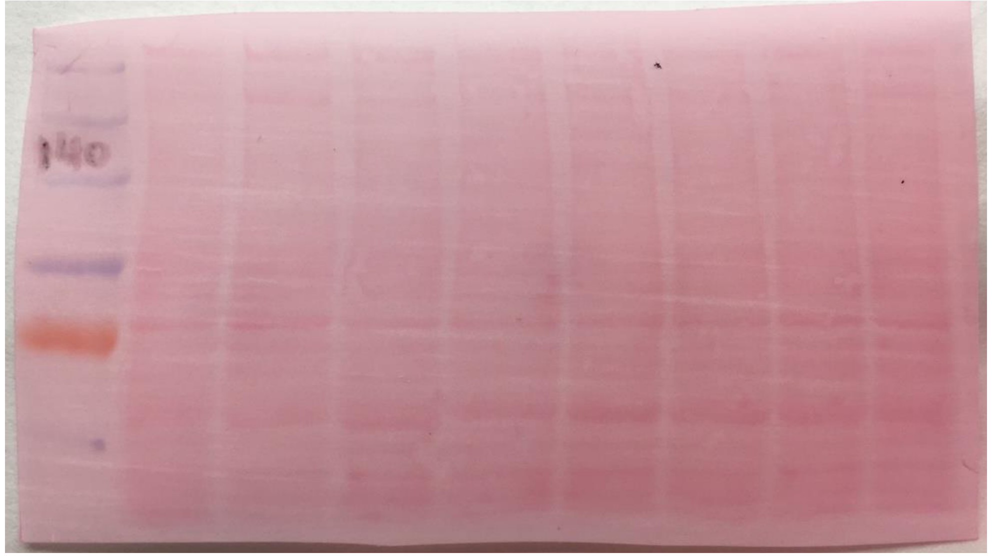

Control Control Imm 7d Imm 7d + Leu

HDAC4
140kDa

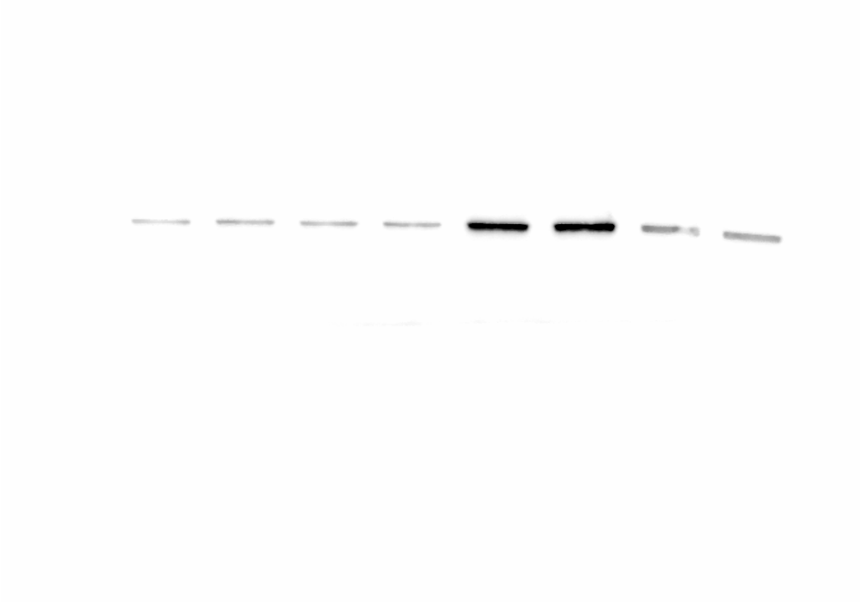

GAPDH
38kDa

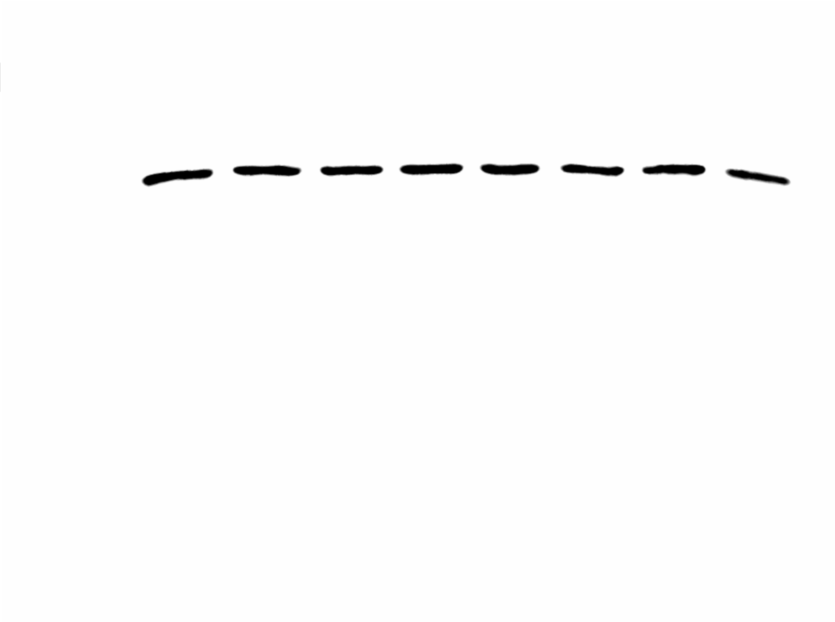

Supplement: Supplementary file 6 — Figure S6. Figures information. [file MUS-72-139-s002.pdf]
