# Supplementary material for: Leucine Supplementation Counteracts the Atrophic Effects of HDAC4 in Rat Skeletal Muscle Submitted to Hindlimb Immobilization
Source: Muscle Nerve. 2025 Apr 4;72(1):139–48. doi: 10.1002/mus.28411 (PMC12138493; doi:10.1002/mus.28411)
Supplement: Supplementary file 8 — Data S1. Supporting Information. [file MUS-72-139-s004.docx]

**Supplementary Materials**

*Immunofluorescence*

Muscle cross-sections were fixed at room temperature in 4% PFA for 10 minutes, washed (3 times for 5 minutes) with TBS-T (tris-buffered saline, 0.5 M NaCl, 50 mM tris-HCl pH 7.4 with 0.3% TRITON X-100). Subsequently, the sections were incubated with a blocking solution (1 hour, 1% bovine serum albumin in TBS-T) and then incubated overnight at 4 ºC with primary antibodies. Primary antibodies were as follows: rabbit anti-HDAC4 (1:250; Cell Signaling, #7628) and rabbit anti-Laminin (1:1000; Sigma, #L9393). After primary antibody incubation, the slides were washed and submitted to the secondary antibody (1:250 Cy3 Donkey Anti-Rabbit, Jackson ImmunoResearch) in blocking solution (1 hour) and washed. The slides were then mounted with coverslips using mounting media containing 4′,6-diamidino-2-phenylindole (DAPI) (cat# H-1200, Vectashield, Vector Labs). Digital acquisitions and the nuclear localization analysis were performed using a fluorescence microscope Axio Scope.A1 (Carl Zeiss Microscopy GmbH, Göttingen, Germany). The nuclei were selected manually, and the percentage of HDAC4 nuclear localization with DAPI was determined using the software ImageJ (v. 1.45s, National Institutes of Health). The quantifications were conducted in a blinded manner, ensuring that the person who quantified was unaware of the experimental groups.

*Western Blotting*

The tissue samples were ground using a liquid-nitrogen-chilled mortar. The generated powder was homogenized in RIPA buffer (1 mM EDTA, pH 7.4, 0.0625% sodium deoxycholate, 0.0625% nonidet P-40, 6.2 mM sodium phosphate, and protease and phosphatase inhibitor cocktail—Thermo Scientific cat#78445). Homogenates were centrifuged (10,000 rpm for 10 minutes at 4 °C), and the supernatant was quantified (Bradford method).

Isolated total protein (25 µg per lane) was loaded into a 10% polyacrylamide gel (SDS-PAGE) and subjected to electrophoresis (60–120 V for 60–120 min). Proteins were transferred to a polyvinylidene difluoride membrane of 0.45 µm (Thermo Fisher Scientific, CAT: 88518, USA) in a semi-dry system (20 V for 180 min). Membranes were blocked with 5% BSA in tris-buffered saline (TBS-T) with Tween (0.5 M NaCl, 50 mM tris-HCl pH 7.4, 0.1% Tween 20) for 1 hour. Subsequently, membranes were washed with TBS-T containing 0.1% Tween, followed by overnight incubation at 4 °C with primary antibodies. Primary antibodies were as follows: rabbit anti-HDAC4 (1:1000; Cell Signaling, #7628); rabbit anti-GAPDH (1:3000; Cell Signaling, #2118); mouse anti-DDK (1:1000; Origene, #TA50011). Then, membranes were incubated with a secondary antibody (1:30,000, goat anti-rabbit peroxidase cat#111035003, Jackson ImmunoResearch) in a solution containing 5% BSA in TBS-T and Tween 0.1% (1 hour at room temperature). Then, the membranes were washed (TBS-T and Tween). Luminata TM (cat#WBLUF0500, Millipore) reagent was used to visualize specific bands, which were analyzed in the Fusion FX5 XT (Vilber Lourmat) imaging system. Variations in loading were normalized by GAPDH.
