# Supplementary material for: Leucine Supplementation Counteracts the Atrophic Effects of HDAC4 in Rat Skeletal Muscle Submitted to Hindlimb Immobilization
Source: Muscle Nerve. 2025 Apr 4;72(1):139–48. doi: 10.1002/mus.28411 (PMC12138493; doi:10.1002/mus.28411)
Supplement: Supplementary file 9 — Table S1. List of genes and primers used for gene expression analysis. [file MUS-72-139-s001.docx]

**Supplementary Materials**

| **Supplemental Table 1:** List of genes and primers used for gene expression analysis. | | | |
| --- | --- | --- | --- |
| **GENE** | **GENE ID** | **Forward 5´-3´** | **Reverse 3´-5´** |
| Atrogin-1 | NM_133521.1 | GAA CAG CAA AAC CAA AAC TCA GTA | GCT CCT TAG TAC TCC CTT TGT GAA |
| HDAC4 | NM_053449.1 | TGG AAG AGC TGC AGA CAG TG | TCA GCG AGC TGT CCA GTT TC |
| Cyclophilin A | NM_017101.1 | TAT CTG CAC TGC CAA GAC TGA GTG | CTT CTT GCT GGT CTT GCC ATT CC |
